# *QuickStats*: Injury Death Rates* for Persons Aged 15–19 Years, by Intent — United States, 1999–2017

**DOI:** 10.15585/mmwr.mm6809a5

**Published:** 2019-03-08

**Figure:**
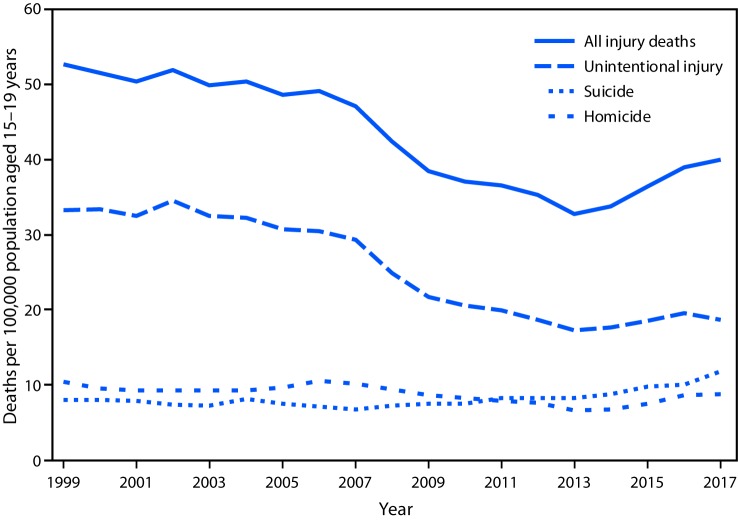
The injury death rate for persons aged 15–19 years declined from 52.7 per 100,000 in 1999 to 32.8 in 2013 but then increased to 40.0 in 2017. Homicide, suicide, and unintentional injury rates have all declined since 1999, with suicide rates beginning to increase in 2008 and homicide rates increasing in 2014. There was not a clear pattern for unintentional injury from 2013 to 2017. Throughout the period, the death rate for unintentional injury was higher than for suicide and homicide, but the difference has narrowed over the past decade. In 2017, the death rate for unintentional injury was 18.7, for suicide was 11.8, and for homicide was 8.7.

**Source:** National Vital Statistics System. Underlying cause of death data, 1999–2017. https://wonder.cdc.gov/ucd-icd10.html.

For more information on this topic, CDC recommends the following link: https://www.cdc.gov/injury/.

